# A COMMUNITY-BASED APPROACH TO REFINING THE ENHANCING ACTIVE CAREGIVER TRAINING (ENACT) INTERVENTION

**DOI:** 10.1093/geroni/igad104.0152

**Published:** 2023-12-21

**Authors:** Jacqueline Eaton, Sarah Neller, Moroni Fernandez Cajavilca, Julene Johnson, Lee Ellington

**Affiliations:** University of Utah, Salt Lake City, Utah, United States; University of Tennessee, Knoxville, Knoxville, Tennessee, United States; New York University, New York City, New York, United States; University of California San Francisco, San Francisco, California, United States; University of Utah, Salt Lake City, Utah, United States

## Abstract

Several interventions for dementia caregivers target the negative effects of behavioral symptoms. Evidence suggests that actively engaging caregivers in training and preparation improves outcomes, such as reduced caregiver burden, depression, and improved subjective well-being. However, there is little information on the best approaches to optimize active engagement. Enhancing Active Caregiver Training (EnACT) is an arts-based intervention that facilitates active engagement using participant-informed vignettes that portray caregiving experiences. This presentation will describe the process of partnering with dementia caregivers to iteratively develop the EnACT intervention in preparation for a randomized controlled trial (RCT). We conducted three iterative focus groups in partnership with dementia caregivers (n=9). Feedback was incorporated into intervention design and materials following each meeting. During focus group one we reviewed and identified video vignettes for inclusion. In focus group two, we tested intervention activities. In focus group three, participants provided feedback on the facilitator guide developed during this process. Focus groups were audio-recorded, transcribed, and analyzed in three cycles using structural, descriptive, and pattern coding. Across all focus groups, we coded 679 items as what went well and 358 items as needing to change. Intervention revisions focused on narrowing vignette topics, removing confusing components (such as engagement activities that were too burdensome), enhancing accessibility, simplifying instructions, and adding facilitator training. A facilitator guide was developed with four main components: Letter to facilitator, Introduction, Preparation to Begin, and Instructions. Partnering with caregivers improved our ability to enhance acceptability and fidelity in preparation for future testing in a wait-list RCT.

